# Characterization of MLST-99 Salmonella Typhimurium and the monophasic variant I:4,[5],12:i:- isolated from Canadian Atlantic coast shellfish

**DOI:** 10.1099/mic.0.001456

**Published:** 2024-05-16

**Authors:** Lisa M. Hodges, Ashley Cooper, Adam Koziol, Catherine D. Carrillo

**Affiliations:** 1 Canadian Food Inspection Agency, Dartmouth, Canada; 2 Canadian Food Inspection Agency, Ottawa, Canada

**Keywords:** *Salmonella*, shellfish, whole-genome sequencing

## Abstract

*Salmonella enterica* subsp. *enterica* Typhimurium and its monophasic variant I 1;4,[5],12:i:- (MVST) are responsible for thousands of reported cases of salmonellosis each year in Canada, and countries worldwide. We investigated *S*. Typhimurium and MVST isolates recovered from raw shellfish harvested in Atlantic Canada by the Canadian Food Inspection Agency (CFIA) over the past decade, to assess the potential impact of these isolates on human illness and to explore possible routes of shellfish contamination. Whole-genome sequence analysis was performed on 210 isolates of *S*. Typhimurium and MVST recovered from various food sources, including shellfish. The objective was to identify genetic markers linked to ST-99, a sequence type specifically associated with shellfish, which could explain their high prevalence in shellfish. We also investigated the genetic similarity amongst CFIA ST-99 isolates recovered in different years and geographical locations. Finally, the study aimed to enhance the molecular serotyping of ST-99 isolates, as they are serologically classified as MVST but are frequently misidentified as *S*. Typhimurium through sequence analysis. To ensure recovery of ST-99 from shellfish was not due to favourable growth kinetics, we measured the growth rates of these isolates relative to other *Salmonella* and determined that ST-99 did not have a faster growth rate and/or shorter lag phase than other *Salmonella* evaluated. The CFIA ST-99 isolates from shellfish were highly clonal, with up to 81 high-quality single nucleotide variants amongst isolates. ST-99 isolates both within the CFIA collection and those isolated globally carried numerous unique deletions, insertions and mutations in genes, including some considered important for virulence, such as gene deletions in the type VI secretion system. Interestingly, several of these genetic characteristics appear to be unique to North America. Most notably was a large genomic region showing a high prevalence in genomes from Canadian isolates compared to those from the USA. Although the functions of the majority of the proteins encoded within this region remain unknown, the genes *umuC* and *umuD*, known to be protective against UV light damage, were present. While this study did not specifically examine the effects of mutations and insertions, results indicate that these isolates may be adapted to survive in specific environments, such as ocean water, where wild birds and/or animals serve as the natural hosts. Our hypothesis is reinforced by a global phylogenetic analysis, which indicates that isolates obtained from North American shellfish and wild birds are infrequently connected to isolates from human sources. These findings suggest a distinct ecological niche for ST-99, potentially indicating their specialization and adaptation to non-human hosts and environments, such as oceanic habitats.

## Data Summary

Genome sequences generated in this study have been submitted to NCBI ENA under study accession numbers PRJEB40107 (RSV-A) and PRJEB40108 (RSV-B). The genome assembly with annotation have also been deposited in the NCBI GenBank under accession numbers OQ941653–OQ941776. The authors confirm all supporting data, code and protocols have been provided within the article or through supplementary data files.

## Introduction

*Salmonella enterica* subsp. *enterica* Typhimurium and its monophasic variant I 1;4,[5],12:i:- (MVST) are responsible for thousands of reported cases of salmonellosis each year in Canada, the USA and many countries worldwide [1,4]. In Canada, *S*. Typhimurium is consistently in the top three serovars implicated in human illness each year, but in 2019, *S*. Typhimurium and MVST ranked the second and third most commonly reported *Salmonella* serovars respectively, marking the first time MVST had ranked in the top three [5], a trend that has also been reported in other areas, such as the EU [4]. Since the emergence of MVST in the 1990s, its prevalence in the environment and association with human illness has been increasing, and in some countries increasing as the prevalence of *S*. Typhimurium declines [6,7].

Transmission of *Salmonella*, including *S*. Typhimurium and MVST, to humans is most often associated with the consumption or handling of eggs, vegetables and land animals (e.g. chicken, pigs and cattle) [8,9]. *Salmonella* including *S*. Typhimurium has also been linked to human illnesses and outbreaks stemming from the consumption or handling of contaminated shellfish (e.g. oysters, clams, mussels and crayfish) [8,10, 11]. Among these, the highest risk is posed by raw shellfish, such as oysters. Survival studies have shown that *Salmonella* are able to survive in aquatic environments for weeks to months, even though they are not naturally found there [12,14]. Contamination of aquatic environments is often due to run-off from agricultural land, sewage discharge and from wild animals (e.g. shoreline birds). Shellfish, specifically bivalve molluscs (e.g. clams, oysters, mussels), are filter-feeders, and as such contaminants, including *Salmonella,* present in the water can begin to accumulate in the tissue, reaching concentrations that can lead to illness when ingested [15]. The impact of the aquatic environment and wild birds or animals on the transmission of *Salmonella* has not been studied to the same extent as other routes of transmission, e.g. domestic animals used in food production or produce [16]. Shellfish surveillance studies have provided insight into the prevalence of *Salmonella* in shellfish, along with the serovars commonly connected with contamination [15,17,19]. However, little work has been focused on the lineages commonly associated with shellfish, their relevance to human illness or the potential routes of contamination.

In this study, we investigated *S*. Typhimurium and MVST isolates recovered from raw shellfish harvested in Atlantic Canada through regulatory testing conducted by the Canadian Food Inspection Agency (CFIA), in comparison to isolates recovered from other food sources. MVST isolates with the multilocus sequence type (MLST) 99 were commonly recovered in shellfish (e.g. oysters and clams) but not from other foods. Furthermore, ST-99 isolates are serologically identified as MVST but almost always identified as *S*. Typhimurium by molecular serotyping (i.e. SISTR tool) [20]. The objectives of this study were (1) to investigate whether recovery of ST-99 from shellfish in Atlantic Canada was potentially due to differences in the growth kinetics of isolates in standard enrichment broth, (2) identify genetic markers which may be unique to ST-99 isolates that may explain their high prevalence in shellfish, (3) to evaluate genomic similarity of shellfish MVST isolates to those recovered from other sources, including human clinical samples and (4) to determine whether a genetic basis could be identified for why ST-99 isolates were not consistently serotyped as MVST.

## Methods

### Bacterial isolates

Bacterial isolates used in this study were collected by the CFIA through routine testing, investigations, environmental monitoring and surveillance studies (Table S1, available in the online version of this article). A total of 210 isolates, recovered from a variety of food products and from environmental swabs between 1998 and 2022, were used in this study. All isolates recovered from shellfish (e.g. oysters, mussels and clams, *n*=133) were harvested along the Canadian Atlantic coast. Isolates were stored cryogenically at −80 °C and resuscitated on tryptic soy agar (TSA) for 24 h at 35 °C. A subset of the strains used in this study (*n*=128 isolates) were previously serotyped at the Public Health Agency of Canada (PHAC) *Salmonella* Reference Laboratory (Guelph, Ontario). Standard methods were used to assess the antigenic formula of each strain [21,22], and serotypes were determined based on the White–Kauffmann–Le Minor scheme [23].

### Comparison of growth in buffered peptone water

A total of 30 *Salmonella* strains were evaluated for growth in buffered peptone water (BPW; Oxoid). The panel consisted of 12 *S*. Typhimurium and MVST strains and 18 strains of non-Typhimurium *Salmonella* (Table 1). The isolates were selected to ensure a genetically diverse panel of *S*. Typhimurium and MVST recovered from shellfish and non-shellfish sources, in addition to non-Typhimurium serovars. Each isolate was streaked onto TSA and grown overnight at 35 °C; a single colony from the plate was then used to inoculate 3 ml of brain heart infusion broth (BHI) which was incubated overnight at 35 °C. The inoculum was prepared by serially diluting the overnight culture in BPW. A 96-well flat-bottom microtitre plate was prepared by adding 20 µl of inoculum to 180 µl of BPW, with each well inoculated with between 10 and 30 c.f.u. Initial c.f.u. counts were determined by drop-plating 50 µl of serially diluted sample onto TSA and incubating for 24 h at 35 °C. The plate was prepared with triplicate wells for each isolate and uninoculated blank media as a control. Using a ClarioStar plate reader (BMG Labtech), the plate was incubated at 35 °C with OD_600 nm_ readings taken every 15 min for 30 h. Each growth trial was performed in triplicate and the length of the lag phase (h) and rate of growth (OD_600_ h^–1^) were estimated using DMFit with a Gompertz curve [24].

**Table 1.** The calculated lag phase and exponential phase rate of change during growth in buffered peptone water

Strain	rMLST*	MLST†	Serotype‡	SISTR§	Source	Rate of change (OD_600_/h)	Lag phase (h)
CFIAFB20130209	1367	19	I 4,5,12:i:-	I 4;[5];12:i:-	Poultry	0.067±0.006	6.99±1.69
CFIAFB20090309	60 228	19	I 4,5,12:i:-	I 4;[5];12:i:-	Poultry	0.064±0.003	6.38±0.74
CFIAFB20140234	1369	34	I 4,12:i:-	I 4;[5];12:i:-	RTE-meat	0.067±0.002	5.96±1.42
CFIAFB20180392	7807	99	I 4,5,12:i:-	I 4;[5];12:i:-	Shellfish	0.074±0.01	7.12±1.13
CFIAFB20150217	7807	99	I 4,12:i:-	Typhimurium	Shellfish	0.063±0.011	7.12±0.64
CFIAFB20150208	7807	99	I 4,12:i:-	Typhimurium	Shellfish	0.068±0.003	7.39±0.38
CFIAFB20160237	7807	99	I 4,12:i:-	Typhimurium	Shellfish	0.068±0.002	7.11±0.08
CFIAFB20180342	7807	99	Typhimurium	Typhimurium	Shellfish	0.062±0.005	6.52±0.55
CFIAFB20120263	26 999	19	Typhimurium	Typhimurium	Shellfish	0.079±0.012	10.75±0.53
CFIAFB20040023	3826	19	Typhimurium	Typhimurium	Feed	0.074±0.007	9.73±0.37
CFIAFB20140159	1344	19	Typhimurium	Typhimurium	Poultry	0.068±0.006	4.93±0.94
CFIAFB20120200	52 648	3719	Typhimurium	Typhimurium	Shellfish	0.059±0.001	6.69±1.21
CFIAFB20150248	26 010	2041	Abaetetuba	Abaetetuba	Sprouted trail mix	0.081±0.002	5.51±0.36
CFIAFB20190115	nd	nd	Adelaide	Adelaide	Pepper	0.093±0.003	6.57±0.55
CFIAFB20140290	3713	22	Branderup	Branderup	Egg swab	0.085±0.004	6.89±0.36
CFIAFB20090229	3721	286	Cubana	Cubana	Sprouts	0.084±0.005	5.72±0.57
CFIAFB20180293	1429	10	Dublin	Dublin	Veal	0.071±0.011	8.1±0.99
CFIAFB20160201	1425	4747	Enteritidis	Enteritidis	Herb	0.076±0.008	6.44±0.2
CFIAFB20140285	7985	239	Gaminara	Gaminara	Flax powder	0.06±0.002	4.13±0.57
CFIAFB20160188	58 450	3780	Glostrup	Glostrup	Peppercorn	0.079±0.011	4.69±0.72
CFIAFB20140389	1428	15	Heidelberg	Heidelberg	Egg wash	0.077±0.004	8.13±0.11
CFIAFB20170236	26 491	32	Infantis	Infantis	Chicken burgers	0.076±0.009	5.21±0.95
2016-HCLON-0003	28 287	2666	Mishmarhaemek	Mishmarhaemek	Bovine	0.086±0.009	5.98±1.07
CFIAFB20100198	1405	316	Montevideo	Montevideo	Tahini	0.069±0.001	4.61±0.26
CFIAFB20120236	8055	2769	Muenchen	Muenchen	Nuts	0.069±0.005	4.73±0.12
2020-MR-Newport	nd	nd	Newport	Newport	Human	0.069±0.008	4.98±1.62
CFIAFB20090265	8040	447	Poona	Poona	Herb	0.089±0.003	6.39±0.54
CFIAFB20180387	8018	96	Schwarzengnund	Schwarzengnund	Shellfish	0.062±0.008	3.64±0.55
CFIAFB20110106	7973	14	I 19:i:-	Senftenburg	Tahini	0.081±0.011	6.91±0.54
CFIAFB20170331	3704	26	Thompson	Thompson	Swab	0.096±0.003	5.65±0.11

a. *Ribosomal multilocus sequence type determined by the 53 -gene scheme developed by Jolley *et al.* [32],; nd=not determined.

b. †Multilocus sequence type based on the 7seven -gene Achtman scheme [31].

c. ‡Serotype determined by antigenic formula based on on the White-–Kauffmann-–Le Minor scheme [23].

d. §Serotype determined using the *Salmonella* In Silico Typing Resource (SISTR) version 1.1.3 [20].

### Whole-genome sequence analysis

All isolates were characterized by whole-genome sequencing (WGS) analysis. Briefly, bacterial isolates were cultured in BHI broth (Oxoid) for 16–24 h at 37 °C, and genomic DNA (gDNA) was extracted using the Maxwell 16 Cell SEV DNA Purification kit (Promega) according to the manufacturer’s instructions. DNA was quantified using the Quant-iT High-Sensitivity DNA Assay Kit (Life Technologies). Sequencing libraries were constructed from 1 ng of gDNA using the Nextera XT DNA Sample Preparation Kit (Illumina) and the Nextera XT Index Kit (Illumina) as recommended by the manufacturer. Paired-end sequencing was performed on the Illumina MiSeq Platform (Illumina) with a 600-cycle MiSeq Reagent kit v3 (Illumina).

The quality of raw sequencing reads was assessed with FastQC version 0.11.8 [22], and quality trimmed with BBDuk from BBTools version 38.22 [25] with the following parameters: trim quality of 10 and removal of reads below 50 bp long. Error correction was performed using tadpole version 8.22 from BBTools in ‘correct’ mode with default parameters. Contigs were assembled from the trimmed and error-corrected reads using SKESA version 2.3.0 with the vector percent argument disabled [26]. Assembly quality was assessed with Qualimap version 2.2 [27,28] and QUAST version 2.3 [29]. Sequences were checked for contamination using ConFindr 0.5.0 with default settings [30].

WGS-based typing was conducted using custom Python scripts (https://github.com/OLC-Bioinformatics/COWBAT/tree/v0.1.5). Multilocus sequence type (MLST) [31] and ribosomal MLST (rMLST) profiles [32] were determined using databases available at https://pubmlst.org/. *Salmonella* serotypes were predicted using the *Salmonella* In Silico Typing Resource (SISTR) version 1.1.3 [20]. The serovar, MLST, rMLST and source information for each isolate are available in Table S1. Single nucleotide variants (SNVs) among strains were identified using SNVPhyl version 1.01 [33].

### Genome-wide association study

A genome-wide association study (GWAS) was performed to identify differences in protein complements between CFIA ST-99 isolates and all other CFIA *S.* Typhimurium and MVST isolates included in this study (Table S1). Roary [34] was used to identify all known and hypothetical proteins, and Scoary [35] was used to calculate the probability that a gene in the accessory genome was specific to either ST-99, the serological profile and/or isolation from shellfish. Confirmation of genetic differences between groups was performed as described in the subsequent section.

### Genomic and proteomic sequence analysis of CFIA genomes

Draft genome assemblies of CFIA *S.* Typhimurium and MVST isolates (*n*=210) were screened for mutations, insertions or deletions in the genomes to identify genetic markers specific to ST-99 or markers specific to the MVST serotype. Genomes were screened for mutations or deletions in flagellar genes previously linked to a monophasic phenotype (i.e. *hin, fljA, fljB* and *fliC*), genes identified through the GWAS specific to ST-99, and changes within the *sseL, safB*, *sthC* and *lpfC* genes which have been reported to be specific to *S.* Typhimurium isolates recovered from water birds (which included ST-99 and ST-3719 isolates) [16,36]. *Salmonella* Typhimurium LT2 (NC_003197.2) was used as a reference genome for gene comparisons where possible and the NCBI non-redundant protein database was used to retrieve sequences for genes not found in the reference genome. GeneSeekr (https://github.com/OLC-Bioinformatics/GeneSeekr) was used to screen for genes in the draft assemblies through BLASTn [37] sequence comparisons against the reference genes, using a minimum percentage identity of 70 % for detection. Alignment and translation of nucleotide sequences were done using Geneious Prime 2019.

### Prevalence of ST-99 genomic features in publicly available databases

All publicly available genome assemblies of *S.* Typhimurium ST-99 (*n*=400) and ST-3719 (*n*=27) in the Enterobase *Salmonella* collection [38] as of January 2023 were screened for many of the genetic features identified as specific to ST-99. A phylogenetic tree was reconstructed from rMLST 7807 genomes within the Enterobase and CFIA collection using R version 3.6.3. All 505 genomes were first analysed using dRep compare [39] to obtain average nucleotide identity (ANI) values between all isolates. A distance matrix was computed and clustered from the Ndb output of dRep using the euclidean and ward methods, respectively. The clustered distance matrix was converted to a dendrogram using the dendextend package [40] and then to a phylo object using the ape package [41], and plotted alongside isolation source and location metadata using the ggtree [42] and ggplot2 [43] packages.

## Results

### Genetic stability of ST-99 and ST-3719 isolates spanning years

**Fig. 1.**
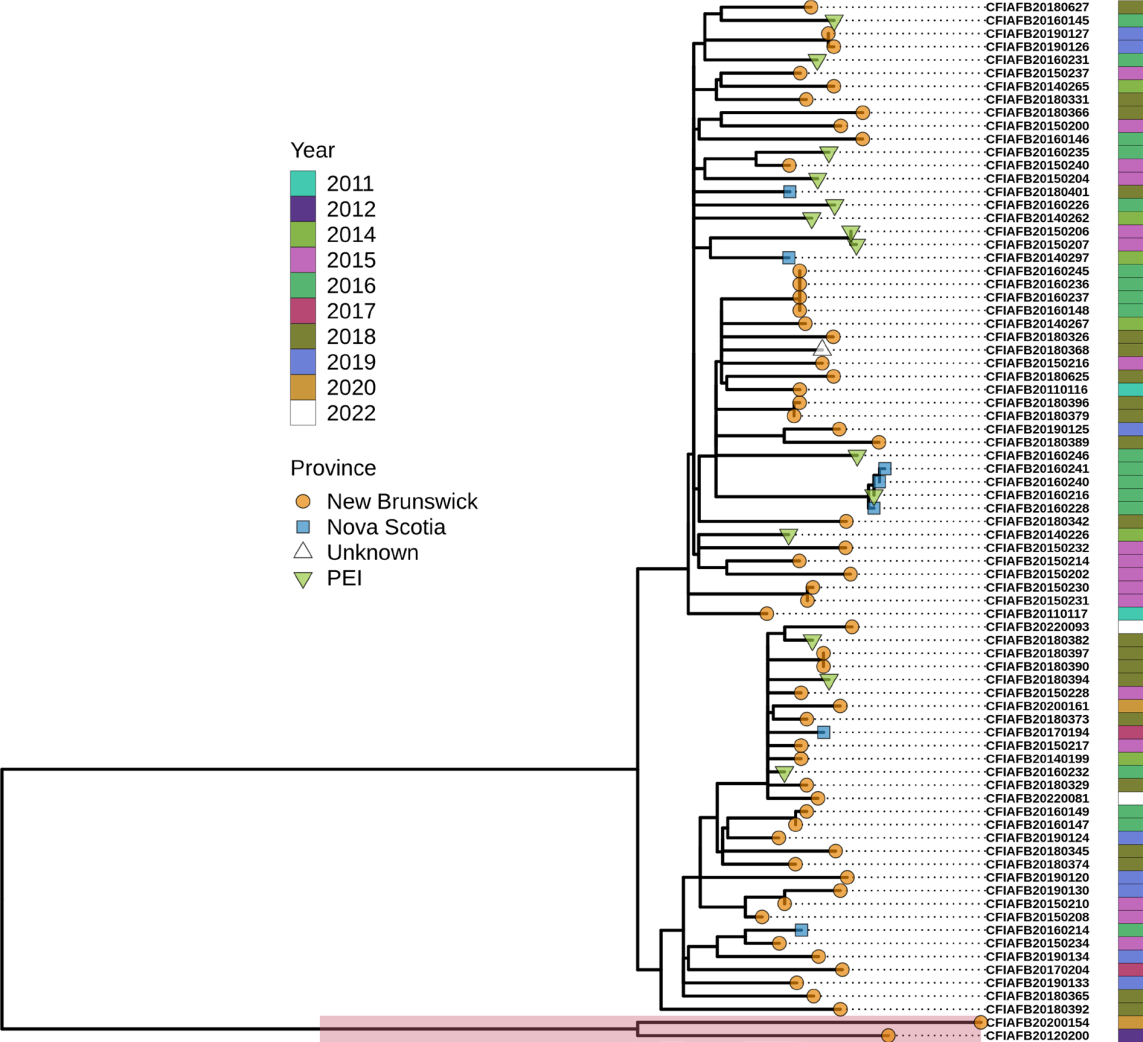
Maximum-likelihood phylogeny of the ST-99 and ST-3719 isolates recovered by the CFIA based on 1420 high-quality SNVs amongst 78 genomes. The complete genome for strain CFIAFB20160237 was used as a reference, and the core genome included 91.5 % of the reference genome used in the analysis. Tip shape indicates province of isolation and colour indicates year of isolation as described in the key. The two ST-3719 isolates are shaded on the dendrogram in red.

To assess clonality of MVST ST-99 isolates collected from shellfish, a phylogenomic analysis based on SNVs identified in the core genome of all ST-99/ST-3719 isolates (*n*=77 and 2 respectively) collected between 2011 and 2022 along the coastal regions of the Maritime provinces (i.e. New Brunswick, Prince Edward Island and Nova Scotia) was done. Isolates with no SNV differences were represented by a single genome. Two ST-3719 genomes, both isolated from shellfish harvested in the same areas as the ST-99 isolates, were included in analysis due to the epidemiological relationship and genetic similarity of these MLSTs, which differ by only one allele and have been previously shown to be closely related [16,36]. There were up to 81 high-quality SNVs between any two of the ST-99 genomes, whereas the ST-3719 genomes had 246–284 SNVs relative to the ST-99 genomes. A maximum-likelihood phylogenetic dendrogram of the ST-99 and ST-3719 isolates (Fig. 1) highlights that the isolates do not appear to cluster according to year or region (i.e. province).

### Growth of *Salmonella* isolates in buffered peptone water

Growth rates of ST-99 MVST were evaluated to assess whether recovery of these organisms, and not other *Salmonella* serovars, from shellfish was due to a selection bias during enrichment culture. All 30 strains of *Salmonella* were able to grow effectively in BPW, but differences between strains were observed in the duration of the lag phase and the exponential growth rate (Table 1). The length of the lag phase varied significantly among strains, with a range from 3.64 to 10.75 h and a median of 6.42 h. Similarly, the rate of the exponential phase also varied across strains and serovars, with the increase in OD_600_ h^–1^ ranging from 0.06 to 0.093 with a median of 0.073. Overall, the length of the lag phase was not associated with exponential growth rate. Strains with a short lag phase did not grow faster during the exponential phase (i.e. the rate of change) and vice versa. For example, 2016-HCLON-0003, serovar Mishmarhaemek, had the shortest lag phase of the strains tested (3.64 h) but was ranked 27^th^ of 30, one of the lowest exponential growth rates calculated (i.e. slowest growth rate). In contrast, S302-3, an MVST, had the longest lag phase of the 30 strains (10.75 h), but had the tenth fastest rate of change. The *S.* Typhimurium and MVST isolates from shellfish did not have a lag phase or exponential phase which was consistently faster or slower than isolates recovered from other sources (poultry, spices, etc.), or other serovars.

### ST-99 isolates lack proteins observed in other *S.* Typhimurium

The GWAS resulted in the detection of several known and predicted proteins with modified amino acid sequences or proteins that were absent in all ST-99 compared to the *S.* Typhimurium reference genome and other *S.* Typhimurium MLSTs within the CFIA collection. The exception to this were three ST-3719 isolates which carried many of the same unique genetic features as ST-99 and had also been isolated from shellfish harvested from areas along the Canadian Atlantic coast (Tables 2 and S1). Three multigene deletions and truncations were found in the ST-99 genomes, two of which were also found in the ST-3719 genomes (Table 2). Of notable absence in both ST-99 and ST-3719 isolates (*n*=117) was the complete deletion of the genes *tae*4, *tai*4 and *hcp*2 (STM0277, STM0278 and STM0279, respectively), located at approximately the mid-region of the type VI secretion system (T6SS) (Table 2). The other two gene clusters spanned STM0721–STM0723 and STM1555–STM1557, identified as primarily putative transporters (e.g. sugar ABC transporter ATP-binding protein, ABC transporter permease), an anti-transporter (e.g. Nhac) and transferases (e.g. glycosyltransferase family 1 protein) (Table 2). In both instances the first and third genes were partially deleted and the second gene in the cluster was completely absent. In all three gene cluster deletions there was no evidence of a recombinant event as in all three instances no DNA was found in place of the deleted DNA.

**Table 2.** Proteins identified by GWAS as being absent, truncated or with a mutation in ST-99 compared to other *S.* Typhimurium and MVST collected by the CFIA

*S*. Typhimurium LT2* gene	Protein†	Function	Change‡	Present in ST-99, ST-3719
**Gene clusters**				
STM0277	Tae4	Type VI secretion system amidase effector protein	Absent	Both
STM0278	Tai4	Type VI secretion system amidase immunity protein	Absent	Both
STM0279	Hcp2	Hcp family type VI secretion system effector	Absent	Both
STM0721		Glycosyltransferase family 1 protein	Partial deletion	ST-99
STM0722		ABC transporter permease	Absent	ST-99
STM0723		Sugar ABC transporter ATP-binding protein	Truncation	ST-99
STM1555		Putative transcriptional regulator	Truncation	Both
STM1556	NhaC	Na^+^/H^+^ antiporter	Absent	Both
STM1557		Putative aminotransferase	Partial deletion	Both
**Single genes**				
*ydhB*	YdhB	Putative LysR family transcriptional regulator	226delA, frameshift	Both
*ytfF*	YtfF	DMT family transporter	123delG, PMSC	ST-99
*rimK*	RimK	30S ribosomal protein S6-l-glutamate ligase	155delG, PMSC	Both
*fidL*	FidL	Hypothetical protein	335delT, PMSC	Both
*ydcX*	YdcX§	Putative inner membrane protein	T23G, PMSC	Both
*sthC*		Putative fimbrial usher protein	1193delC	Both
*safB*	SafB	Putative fimbriae assembly chaperone	597insC	
STM0148		Alpha-*N*-arabinofuranosidase	448delA, PMSC	Both
STM2287	SseL	Putative cytoplasmic protein	467insA, PMSCT816G	Both
STM2361		Putative regulatory protein	G1088A	Both
STM3530	NicT	Pseudogene, putative permease	C507G, PMSC	Both
STM3774		Putative inner membrane protein	172delA, PMSC	Both

a. *Accession NC_003197.2.

b. †Protein identification based on genome annotation and NCBI non-redundant protein database.

c. ‡Positions and substitutions are based on coding sequence; PMSC=premature stop codon.

d. §Identified as GhoT/OrtT family toxin in NCBI non-redundant protein database.

In addition to these multigene clusters, several genes containing base pair substitutions, insertions or deletions were identified. These mutations led to either a premature stop codon (PMSC) or a frameshift (Table 2). Almost all of the genetic differences identified between ST-99 genomes and the reference genome were also found in the three ST-3719 genomes, with the exception of *ytfF* and a putative protein, for which variants were only found in the ST-99 isolates (Table 2). The proteins identified by the GWAS and by recent studies as being modified or no longer functional were associated with a wide range of known or putative functions, including membrane proteins (e.g. *fidL, ydhX, sthC* and *ytfF*), translation (e.g. *rimK*), transcription (e.g. *ydhB*) and virulence (*sseL*, *safB* and *lpfC*). While all ST-99 and ST-3719 genomes carried the *lpfC* single base deletion, it was not specific to these sequence types as two ST-19 *S.* Typhimurium isolates were also found to carry this mutation, one recovered from an environmental swab of an egg facility and the other recovered from produce (Table S1).

### Genomic feature unique to ST-99 isolates

**Fig. 2.**
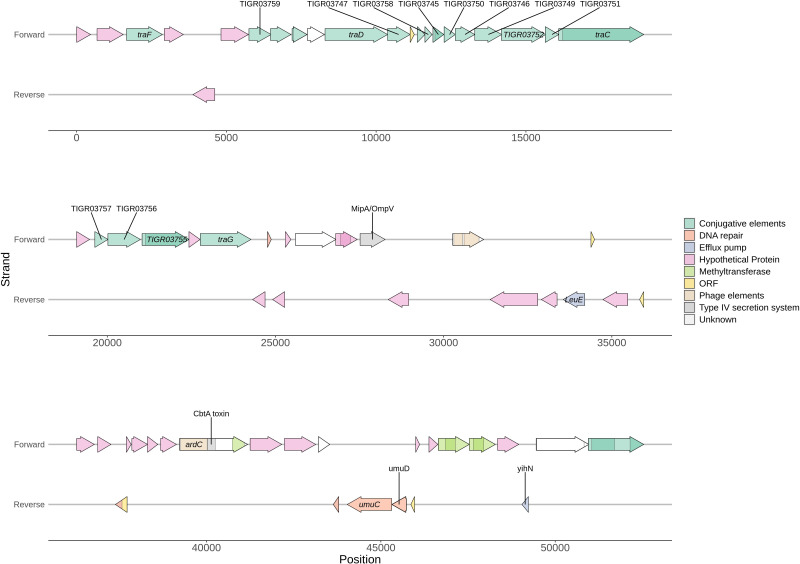
An annotation of 51.2 kb of the approximately 81.1 kb genomic insert found in many of the North American isolates (*n*=124/132). This region of the insert was used for screening all publicly available Enterobase ST-99 and 3719 genomes.

The GWAS conducted with all of the CFIA *S*. Typhimurium and MVST genomes led to the identification of a number of known and hypothetical proteins that were unique to ST-99 isolates and, in most instances, also to the three ST-3719 isolates (Table 3). The identified genes were found within a large genomic insert, spanning approximately 81.1 kb. Most of the region (approx. 52.5 kb; Fig. 2) contains a group of genes primarily coding for proteins of unknown function, although genes associated with stress response, DNA repair and efflux pumps were identified (Table 3). These included genes encoding UmuC and UmuD, the leucine efflux pump Leu, an MipA/OmpV family protein and methyltransferases (Table 3 and Fig. 2). Interestingly, genomes of ST-3719 isolates carried almost the same 52.5 kb region, except for a cluster of four genes, *Leu* and three genes encoding proteins of unknown function, which were absent from the midpoint of the region (Table 3). The UV repair genes *umuC* and *umuD* identified within this genetic region are genetically distinct from the *umuC* and *umuD* genes found in the reference genome and CFIA *S.* Typhimurium and MVST genomes screened. Screening of the *umuC* and *umuD* genes common to all of the *Salmonella* in the CFIA panel found an H338Y substitution (nucleotide C1012T) in the *umuC* of all ST-99 and 3719 isolates. Five additional genes identified by the GWAS in the remaining third of the region were specific to both ST-99 and 3719, of which four encoded hypothetical proteins and the fifth protein was identified as a TraR/DksA C4-type zinc finger protein.

**Table 3.** Genomic region identified by Roary in all ST-99 isolates recovered by the CFIA (*n*=99) and in a proportion of MLST-99 non-CFIA isolates (*n*=131/400). The genomic region, with the exception of the genes in bold type, was also identified in all MLST-3719 isolates.

Gene	NCBI accession no.*	Function
group_377	WP_222985072.1	TraI domain-containing protein
YihN		Hypothetical protein
group_1332	WP_023208053.1	Dienelactone hydrolase family protein
YjhP	WP_023208054.1	Class I SAM-dependent methyltransferase
COQ3	EAR4810783.1	Class I SAM-dependent methyltransferase
group_2101	WP_015606341	TraI domain-containing protein, ref seq
UmuD	WP_023226847.1	Translesion error-prone DNA polymerase V autoproteolytic subunit
UmuC	WP_071953562.1	Y-family DNA polymerase
group_2104	WP_001222413.1	Hypothetical protein
group_2105	WP_216746255.1	DUF3085 domain-containing protein
group_2106	WP_000022217.1	Hypothetical protein
group_2107	WP_015606332.1	ssDNA-binding domain-containing protein
group_2108	WP_071953566.1	Zincin-like metallopeptidase domain-containing protein
**group_2114**	**EAA8759369.1**	**Hypothetical protein AH782_10260, *Salmonella enterica* subsp. Enterica**
**Leu**	**WP_070804378.1**	**Leucine efflux protein**
**group_2116**	**WP_097336870.1**	**Hypothetical protein**
**group_1335**	**WP_001406333.1**	**Hypothetical protein**
mipA	WP_057521402.1	MipA/OmpV family protein
group_1313	WP_225807894.1	Hypothetical protein
group_2054	WP_071953580.1	Hypothetical protein [*Salmonella enterica*]
group_852	WP_057520021.1	TIGR03756 family integrating conjugative element protein
group_2065	WP_000214158.1	Hypothetical protein
group_2066		Hypothetical protein
group_2067	WP_000193306.1	Hypothetical protein
group_2068	WP_071953595.1	Conjugal transfer protein TraF *S. enterica*
group_2069	WP_071953597.1	Hypothetical protein *S. enterica*

a. *NCBI RefSeq non-redundant sequences are denoted by WP_*.

### Prevalence of unique genetic features in MLST-99 can vary globally

Both ST-99 (*n*=400) and ST-3719 (*n*=27) genomes published in Enterobase were screened for the genes identified in the GWAS analysis of the CFIA ST-99 and/or ST-3719 isolates (Table S1B and C). Overall, the majority of the ST-99 genomes were from isolates collected in North America (62.5 %, *n*=250/400) and 74.8 % of all genomes were from isolates recovered from birds, aquatic animals (e.g. fish and shellfish) or river/water sources (Table S1B). All ST-3719 isolates were recovered from wild birds in the USA (Table S1C). Many of the genetic features identified in the CFIA ST-99/3719 genomes (Tables2  3) were also present in all or almost all of the Enterobase genomes (Table S1B and C). For example, the amino acid substitution in *umuC* and mutations in the *fdlL, ydhB, ydcX, lpfC, sseL, sthC* and *rimK* genes were common to both ST-99 and 3719 genomes, consistent with the findings from the GWAS. Deletion of STM1555–STM1557 [includes the putative Na^+^/H^+^ antiporter gene (NhaC)], deletion of the three T6SS genes (i.e. *tae*4, *tai*4 and *hcp2*), and mutations in STM3530 (*nicT*) and *safB* were found in ≥99.5 % of the isolates (i.e. *n*≥398/400 genomes). The absence of these mutations was primarily associated with a North American isolate recovered from an avian source (HB9449AA) which did not carry the mutation in STM3530 (*nicT*) or multi-gene deletions in the T6SS and STM1555 to STM1557. In addition to the mutations screened for, other mutations were identified in the genomes obtained from Enterobase. For example, in two genomes (SAL_FB3463AA and SAL_FB3483AA) the STM2361 substitution which led to a PMSC was found but they were also found to carry a 6 bp insert downstream.

In contrast, the single base deletion in the *yftF* gene, the multigene deletion and truncations of STM0721–STM0723, and the presence of a large genome insertion were identified almost exclusively in genomes from isolates recovered in North America, with the highest prevalence in Canadian isolates. Overall, there was a high likelihood of carrying all three of these genetic features as 72 % of isolates with at least one feature were carrying all three (*n*=132/184) (Table S1). The presence of the large genomic region in ST-99 and ST-3719 was screened using 52.5 kb of the 5′ end and against the individual genes identified by Roary within it. The full-length, 81.1 kb genomic region could not be screened for as a protein identified as a phage tail identified by blast led to misalignments between genomes assembled by different contributing laboratories (Fig. 2). The ST-99 genomic region was only detected in ST-99 isolates recovered in North America, with 52 % (*n*=130/250) of the isolates carrying the full 52.5 kb region. The highest prevalence, however, was in isolates from Canada, with all but three isolates positive for the region (92 %, *n*=36/39) compared to the USA where 46.5 % (*n*=93/200) of isolates were positive (Table S1). All 27 ST-3719 genomes, all from the USA, carried the same gene complement as the three ST-3719 CFIA isolates (i.e. the genomic region of ST-99 but with the absence of four genes), with the exception of one genome. In genome KC3669AA a hypothetical protein was only partially identified, due to a deletion of 99 bases from the mid-point of the gene (Table S1). The highest prevalence of the insert in ST-99 genomes was in isolates recovered from shellfish (*n*=20/23), followed by birds (*n*=68/131), although this may reflect the fact that a large proportion of the Enterobase genomes used in this study were reportedly from these two sources (39 %, *n*=155/400). All of the genomes from isolates reportedly from shellfish carrying the insert were isolated from Canada whereas the majority of avian isolates were from the USA.

The deletion within STM0721–STM0723 was the most variable mutation observed in the ST-99 genomes from Enterobase. Overall, almost all isolates with a complete deletion of STM0722 (i.e. putative ABC transporter permease) also carried the same partial deletion of STM0721 and STM0723 (*n*=145/147) as identified in the CFIA isolates. All but four of the 150 isolates with this specific multigene deletion were isolated in North America, while the remaining four were isolated in France, reportedly from humans (Table S1). Mutations other than those identified by the GWAS conducted using isolates collected by the CFIA were found in isolates from other regions. These included the complete deletion of all three genes, disruption in one of the genes (e.g. gene split onto two contigs) or partial deletion of only one gene. In contrast, 61 % (*n*=245/400) of the ST-99 genomes in Enterobase did not carry any mutation in STM0721–STM0723 or the genomic insert genes.

### Phylogenetic analysis of CFIA shellfish-associated MVST and published genomes

**Fig. 3.**
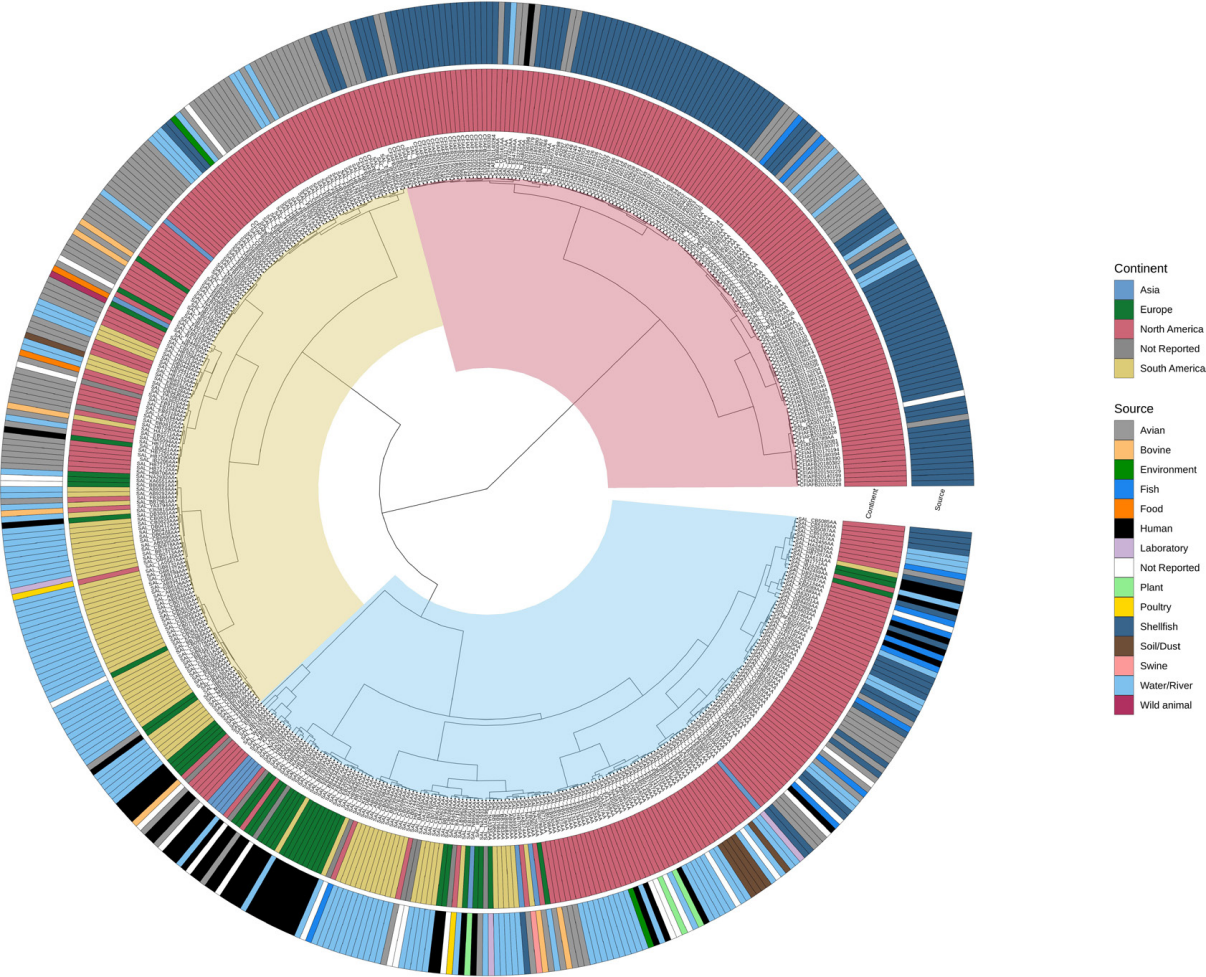
Phylogenetic comparison of all CFIA and publicly available Enterobase rST-7807 genomes. The three major clades are highlighted: red, clade I; yellow, clade II; and blue, clade III. The region and source of the isolate are identified in inner and outer labels, respectively.

As the majority of CFIA and Enterobase ST-99 isolates were identified as rST-7807, a phylogenetic comparison was done to examine the relationship between isolates from different regions and sources, and whether possible links to human clinical isolates could be inferred. A comparison of genomes recovered globally found that genomes were more likely to cluster by region than by the source of isolate (Fig. 3). The genomes branched into three main clades, with increasing diversity in the region and source that the isolate came from. Clade I comprised only genomes from North America, of which the majority were isolated from shellfish and wild birds. Within this clade there was a single genome from a human which closely aligned to genomes from shellfish, wild birds and water. In contrast, clades II and III comprised genomes from multiple regions and sources (Fig. 3). In clade II seven genomes from humans (all from Europe) aligned closely with isolates recovered from a water source and in a few cases to isolates from wild birds. The highest prevalence of genomes from humans was in clade III, which also contained the greatest diversity in the reported source of the isolate. Human-source genomes not only aligned to shellfish and wild bird genomes as seen in the other two clades, but also to isolates from plants, poultry and bovine. Due to the considerably smaller number of genomes from areas outside of North America, it is difficult to reliably infer possible transmission routes for isolates recovered from humans. For example, only 11 of the 43 genomes recovered in Europe were not recovered from a human clinical sample, of which only six had an isolation source identified. Interestingly, no human isolates were found in Enterobase from South America, even though it was the second most represented region. The low number of rST-7807 genomes from these regions may reflect an unknown reservoir of ST-99, differences in reporting of clinical cases or less targeted sampling (e.g. in contrast to shellfish testing by the CFIA or large-scale research projects targeting wild bird populations in the USA).

**Fig. 4.**
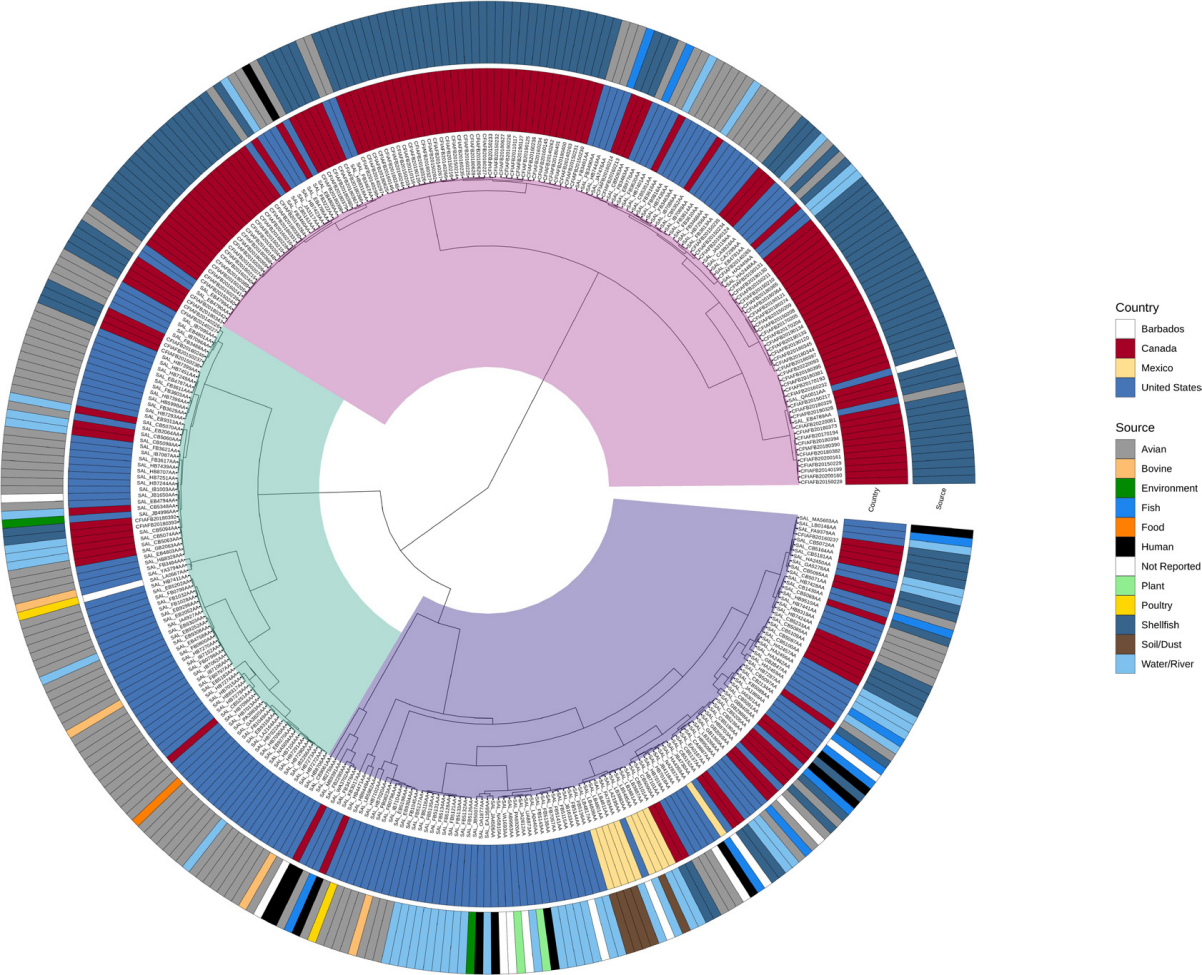
Phylogenetic comparison of all North American rST-7807 genomes, including CFIA and Enterobase genomes. The three major clades are highlighted: pink, clade I; green, clade II; and purple, clade III. The region and source of the isolate are identified in inner and outer labels, respectively.

A phylogenetic analysis of genomes from North America was done to evaluate the relatedness of genomes from different sources in the same region; the majority of isolates were from Canada and the USA, with only 11 of 251 genomes recovered from Mexico and Barbados. Similar to the phylogenetic tree of all genomes, there appeared to be three major clades (Fig. 4), with increasing the diversity of the reported sources of isolates. For example, in clade I the majority of isolates were from Canadian shellfish, followed by isolates from wild birds in the USA, with the exception of several isolates from water sources and one Canadian human isolate (SAL_FB3222AA). The single human isolate formed a small cluster with a Canadian shellfish isolate and with five US isolates from water and birds (Fig. 4). Clade II comprised primarily isolates from wild birds in the USA, with a smaller percentage of genomes from water and Canadian shellfish. No genomes from humans were found in this clade, but it contains single representative genomes from other sources (e.g. poultry, bovine and food). In contrast to the first two clades, clade III contained genomes from isolates recovered from a wide range of sources, including plants, poultry, fish and bovine, and was the clade where nine of the ten North American human isolates clustered. The human isolates were dispersed throughout clade III, and while a proportion of these isolates formed branches with isolates from water, shellfish and birds as was seen in clade I, three human isolates were closely related to isolates from fish, while another aligned closely to one isolated from plants.

### Inconsistent changes observed in flagellar genes across all *S.* Typhimurium

WGS-based determination of serovar using SISTR with 210 draft genomes was generally poor at identifying isolates determined to be MVST using serological approaches, with most being identified as Typhimurium and only 10 of 81 MVST isolates being correctly identified (Table S1A). Sequence analysis of the four flagellar genes (i.e. *hin, fljA, fljB* and *fliC*) was conducted to identify genetic markers that could be used to accurately distinguish MVST or *S*. Typhimurium isolates using draft assemblies (Table S1A). While sequence variants and deletions were identified in each gene, none were consistently associated with any of the monophasic phenotypes (e.g. I 4;[5];12:i:-) with or without the presence of an O antigen (i.e. rough) (*n*=79) or with isolates serologically identified as MVST but misidentified as *S*. Typhimurium by SISTR (*n*=69). For example, screening of the *fljB* gene found gene deletions ranging from 52 to 447 bp from the 3′ or 5′ end of 16 isolates (data not shown) and nine in which the presence of *fliB*, of any length, was not found in the genome assembly. Of these, eight of the nine isolates with no *fljB* detected were both serologically typed and genotyped as MVST; the remaining isolate was serologically typed as *S.* Typhimurium but genotyped as MVST. In contrast, a partial deletion of the *fljB* gene did not impact the serological typing as ten of the 16 isolates were both serologically and genotypical of *S.* Typhimurium, indicating that a full-length *fliB* was not required for the presentation of a biphasic phenotype. In support of this, 63 isolates which had been serologically identified as MVST showed no changes in base substitutions, insertions or deletions in *fljB* compared to the reference genome. Similarly, for the *fliC* gene, full or partial gene deletion or a pE260K substitution did not correlate to a serological or sequence-based determination of MVST or *S*. Typhimurium. In addition, none of the mutations or deletions observed in the *fljB* or *fliC* genes were associated with a specific MLST, rMLST or isolation source (Table S1A).

In contrast, while no deletion or amino acid substitution in the *fljA* and *hin* genes corresponded to a specific serological or predicted serotype (Table S1A), ST-99 was the only MLST in which an *fljA* A46T and Hin R140L substitution were detected (*n*=70). Ido *et al.* [44] reported these mutations as indicative of monophasic variants, but the presence of these two mutations may be a more accurate predictor of monophasic phenotype ST-99.

## Discussion

In this study, we investigated ST-99 isolates recovered from shellfish harvested along the Canadian Atlantic coast to gain potential insight into why this sequence type has only been recovered from raw shellfish (i.e. oysters, clams and mussels) during routine testing by the CFIA. The findings from this study have shown that the exclusive isolation of ST-99 from shellfish is probably not due to faster growth in the enrichment broth used for recovery of *Salmonella* from foods (i.e. BPW) as ST-99 isolates did not consistently have a faster growth rate and/or shorter lag phase than other *Salmonella* evaluated (Table 1). The limited genetic variation observed in the population over 10 years (Fig. 1) suggests that this ST-99 lineage is a persistent population along the Atlantic coastline, rather than sporadically occurring. It is therefore likely that the contamination of shellfish by this sequence type is due to the ability of these isolates to persist longer in aquatic environments compared to other sequence types, possibly reflecting a wild animal or bird host reservoir which inhabits the coastal region or both.

To date, little work has been published regarding the ST-99 lineage, at least in part due to the lower prevalence of the sequence type in surveillance of foods more commonly associated with human illness (e.g. food production animals such as pigs and chicken). Recent studies have indicated that ST-99 is more likely to be associated with water systems, aquatic animals and wild birds than with other sources [16,36]. It is possible that genetic variations found to be common in ST-99 strains reflect host or environmental adaption. The findings from this study support this as water, shellfish and wild birds (e.g. cormorants) were the most common source of ST-99 isolates and genomes from these sources often clustered together (Fig. 3). A recent study by Fu *et al.* [16] reported that in a study of *S*. Typhimurium from wild birds in the USA, the second most common sequence type was ST-99. It was also the only sequence type which was specific to one lineage of birds, water birds, and all of the other sequence types were found distributed across the larid, passerine and water bird lineages. The authors also reported that ST-99 was found to align closely with shellfish and water isolates and rarely with domesticated animals used in food production. Although found primarily in aquatic environments and wild birds, 12 % of the genomes used in this study from Enterobase were recovered from humans, indicating that ST-99 strains are capable of infecting humans. Interestingly, the majority of human isolates were associated with a single clade of the phylogenetic trees (Figs3  4), the clade that also contained the highest degree of source diversity compared to the rest of the tree. It is possible that ST-99 strains associated with humans are more likely to either come from a host other than wild birds or to have an intermediate source other than shellfish or water. Overall, it appears that the strains circulating in North American shellfish and wild birds are the least likely to be recovered from humans.

The GWAS conducted in this study led to the discovery of numerous differences in the genomes of ST-99 compared to other *S.* Typhimurium, though some traits appear to be shared with ST-3719 isolates. These two sequence types appear to be closely related, sharing similar hosts and habitats, as supported by phylogenetic comparisons made in this study and by Fu *et al.* [36], in which the two sequence types cluster together in the tree. The genomes of ST-99, collected by the CFIA and globally, were also found to carry several deletions and mutations in genes considered important for virulence, supporting the hypothesis that ST-99 have become either host-adapted or have reduced pathogenicity. In particular, the complete absence of three key genes from the T6SS and the disruption of genes associated with fimbriae formation (e.g. *sthC* and *safB*) are of particular significance. The T6SS is a system used by Gram-negative bacteria to deliver toxins to eukaryotic or prokaryotic cells. In *Salmonella* spp., *hcp* genes form hexamers which form the inner tube of the contractile tail, while *tae4* and *tai4* code for a peptidoglycan amidase effector (toxin) and the cognate immunity protein, respectively [45,46]. Deletions in or of the T6SS, including *hcp2* and *tae4*, have been shown to have a significant impact on the ability of *S*. Typhimurium to colonize a host [47,48] and reduce the effectiveness of killing common commensal gut bacteria [47], and some studies have found that mutants were less able to colonize the spleen and liver of mice [49]. In fact, studies using mouse models to compare the virulence of strains have shown that the ST-99 strains have not resulted in mortality compared to other sequence types, even 12 days post-infection [50,51]. Proteins involved in the regulation and transportation of substrates, and the cell membrane were also found to be deleted or mutated; however, the possible impact of the mutations on the virulence of ST-99 isolates would be difficult to predict.

Interestingly, not all of the genetic changes found common to the CFIA isolates were detected in the genomes available in Enterobase, suggesting that while there are genetic markers which are characteristic of ST-99, some of the mutations appear to be more representative of a region rather than of a sequence type. One of the most notable findings of this study was the identification of a large genomic region found exclusively in North American isolates, with the highest prevalence being in genomes from Canadian isolates, i.e. 92 % of Canadian isolates carried the insert (*n*=36/39) compared to 47 % of isolates from the USA (*n*=92/197). While the origin of the region is not known, it appears that at least a large portion of this region is also shared by North American ST-3719 isolates. The majority of this region contains proteins of unknown functions; however, of potential interest are the genes with sequence similarity to *umuC* and *umuD*, both of which have been shown to be protective against lethal frameshift mutations, while increasing mutagenesis by UV light and other chemicals in *Salmonella* and closely related bacteria [52,54]. While *S.* Typhimurium does possess these genes, their activity is comparatively lower than those carried by other organisms such as *Escherichia coli* [55]. This reduced efficacy may be attributed to differences in the amino acid sequence of *umuC*. Although the genes identified in this study are genetically distinct from those ubiquitously found in *S.* Typhimurium, and their functionality remains untested experimentally, they may provide additional protection against UV light damage from sunlight exposure. This possibility hints at an adaptive mechanism for environmental survival.

In this study we determined that MVST isolates were frequently misidentified as S. Typhimurium by the genoserotyping tool used, SISTR [20]. Similarly, the genoserotying analyses conducted in Enterobase [38] and provided in the strain metadata indicate that almost all ST-99/ST-3719 are identified as serovar Typhimurium with both SeqSero2 [56] and SISTR (Table S1). No serotype determination was provided by SeqSero for approximately 14 % of MVST genomes in cases where the O-antigen was not identified. The identification of MVST by SISTR for a small number of strains in the present study may be attributed to the use of different versions of the software. While genetic mutations in the flagella genes could not be used to improve the identification of MVST typed as *S.* Typhimurium by SISTR, the study did show that all ST-99 isolates carried the amino acid substitutions in FljA and Hin proteins, variations first described by Ido *et al.* [44]. The authors compared the phase variation frequency in two S. Typhimurium strains into which one or both of these point mutations were introduced. The authors found that the presence of any one mutation decreased the frequency of phase variation and the presence of two mutations resulted in a monophasic profile. Based on this study and Ido *et al.* [44], it is very likely that all ST-99 isolates will be phenotypically MVST, and as such the presence of these mutations should be considered for inclusion in tools used for genoserotyping MVST.

It is important to note that not all MVST genomes displayed the amino acid substitutions in FljA/Hin, highlighting the need for further exploration of genomic elements to refine the accuracy of genoserotyping. Additionally, full and partial deletions of the *fljB* gene were observed in multiple non-ST-99 MVST genomes, a phenomenon also reported by Ido *et al.* [44], suggesting its potential impact on flagellar expression. The likelihood of genomic rearrangements affecting flagellar phase variation cannot be overlooked. Although short-read sequencing platforms, like Illumina, provide efficient and cost-effective genomic analysis, their capability to identify complex structural variations, such as rearrangements and inversions, is limited. Therefore, the integration of long-read sequencing technologies is advocated to achieve a more detailed genomic understanding, thereby enhancing the identification of genetic diversity and its significance for flagellar phase variation in *Salmonella*. Our findings indicate that ST-99 (and probably ST-3719) associated with shellfish are a unique group of MVST, characterized by numerous genetic mutations and, in some geographical regions, a large genetic insertion. Even though the impact of these mutations and insertions was not investigated during this study it can be inferred that these isolates are more adapted for surviving in the environment, such as in ocean water, with wild birds and/or animals as a natural host. This study also indicates that this lineage may have reduced pathogenicity, with very few clinical isolates in North America associated with ST-99 lineages commonly recovered from shellfish.

## supplementary material

10.1099/mic.0.001456 Table S1.

## Abbreviations

ANI: average nucleotide identity
BHI: brain heart infusion
BPW: buffered peptone water
CFIA: Canadian Food Inspection Agency
gDNA: genomic DNA
GWAS: genome-wide association study
MLST: multilocus sequence type
MVST: monophasic variant I 1;4,[5],12:i:-
PMSC: premature stop codon
rMLST: ribosomal multilocus sequence type
SISTR: *Salmonella* In Silico Typing Resource
SNV: single nucleotide variant
TSA: tryptic soy agar
T6SS: type VI secretion system
WGS: whole genome sequencing

## References

[1] Balasubramanian R, Im J, Lee J-S, Jeon HJ, Mogeni OD (2019). The global burden and epidemiology of invasive non-typhoidal *Salmonella* infections. Hum Vaccin Immunother.

[2] Government of Canada (2020). Canadian Integrated Program for Antimicrobial Resistance Surveillance (CIPARS) 2018: Design and Methods.

[3] Medalla F, Gu W, Friedman CR, Judd M, Folster J (2021). Increased incidence of antimicrobial-resistant nontyphoidal *Salmonella* infections, United States, 2004-2016. Emerg Infect Dis.

[4] European food safety authority, European centre for disease prevention and control (2021). The European Union one health 2019 Zoonoses report. EFSA Journal.

[5] Government of Canada (2020). National Enteric Surveillance Program Annual Summary 2019.

[6] Mather AE, Phuong TLT, Gao Y, Clare S, Mukhopadhyay S (2018). New variant of multidrug-resistant *Salmonella enterica* serovar Typhimurium associated with invasive disease in immunocompromised patients in Vietnam. mBio.

[7] Gymoese P, Sørensen G, Litrup E, Olsen JE, Nielsen EM (2017). Investigation of outbreaks of *Salmonella enterica* serovar Typhimurium and its monophasic variants using whole-genome sequencing, Denmark. Emerg Infect Dis.

[8] Kumagai Y, Pires SM, Kubota K, Asakura H (2020). Attributing human foodborne diseases to food sources and water in Japan using analysis of outbreak surveillance data. J Food Prot.

[9] Williams MS, Ebel ED (2022). Temporal changes in the proportion of *Salmonella* outbreaks associated with twelve food commodity groups in the United States. Epidemiol Infect.

[10] Jansson Mörk M, Karamehmedovic N, Hansen A, Nederby Öhd J, Lindblad M (2022). Outbreak of *Salmonella* Newport linked to imported frozen cooked crayfish in dill brine, Sweden, july to november 2019. Euro Surveill.

[11] Venkat H, Matthews J, Lumadao P, Caballero B, Collins J (2018). *Salmonella enterica* serotype javiana infections linked to a seafood restaurant in Maricopa county, Arizona, 2016. J Food Prot.

[12] Topalcengiz Z, McEgan R, Danyluk MD (2019). Fate of *Salmonella* in central florida surface waters and evaluation of EPA worst case water as a standard medium. J Food Prot.

[13] El Mejri S, El Bour M, Boukef I, Al Gallas N, Mraouna R (2012). Influence of marine water conditions on *Salmonella enterica* serovar Typhimurium survival. J Food Saf.

[14] Tamber S, Montgomery A, Eloranta K, Buenaventura E (2020). Enumeration and survival of *Salmonella enterica* in live oyster shellstock harvested from Canadian waters. J Food Prot.

[15] Chakroun I, Fedhila K, Mahdhi A, Mzoughi R, Saidane D (2021). Atypical *Salmonella* Typhimurium persistence in the pacific oyster, *Crassostrea gigas*, and its effect on the variation of gene expression involved in the oyster’s immune system. Microb Pathog.

[16] Fu Y, M’ikanatha NM, Lorch JM, Blehert DS, Berlowski-Zier B (2022). *Salmonella enterica* serovar Typhimurium isolates from wild birds in the United States represent distinct lineages defined by bird type. Appl Environ Microbiol.

[17] Rubini S, Galletti G, D’Incau M, Govoni G, Boschetti L (2018). Occurrence of *Salmonella enterica* subsp. *enterica* in bivalve molluscs and associations with *Escherichia coli* in molluscs and faecal coliforms in seawater. Food Control.

[18] Lopatek M, Wieczorek K, Osek J (2022). Prevalence and antimicrobial resistance of bacterial foodborne pathogens isolated from raw bivalve molluscs subjected to consumption in Poland during a ten-year period. Foods.

[19] Lo Y-T, Wang C-L, Chen B-H, Hu C-W, Chou C-H (2017). Prevalence and antimicrobial resistance of *Salmonella* in market raw oysters in Taiwan. J Food Prot.

[20] Yoshida CE, Kruczkiewicz P, Laing CR, Lingohr EJ, Gannon VP (2016). The *Salmonella* in silico typing resource (SISTR): an open web-accessible tool for rapidly typing and subtyping draft *Salmonella* genome assemblies. PLoS One.

[21] Shipp CR, Rowe B (1980). A mechanised microtechnique for *Salmonella* serotyping. J Clin Pathol.

[22] Andrews S (2010). FastQC: a quality control tool for high throughput sequence data. http://www.bioinformatics.babraham.ac.uk/projects/fastqc.

[23] Grimont PA, Weill F-X WHO Collaborating Centre for Reference and Research on Salmonella.

[24] Baranyi J, Roberts TA (1994). A dynamic approach to predicting bacterial growth in food. Int J Food Microbiol.

[25] Bushnell B (2014). BBMap: a fast, accurate, splice-aware aligner. https://www.osti.gov/servlets/purl/1241166.

[26] Souvorov A, Agarwala R, Lipman DJ (2018). SKESA: strategic k-mer extension for scrupulous assemblies. Genome Biol.

[27] García-Alcalde F, Okonechnikov K, Carbonell J, Cruz LM, Götz S (2012). Qualimap: evaluating next-generation sequencing alignment data. Bioinformatics.

[28] Okonechnikov K, Conesa A, García-Alcalde F (2016). Qualimap 2: advanced multi-sample quality control for high-throughput sequencing data. Bioinformatics.

[29] Gurevich A, Saveliev V, Vyahhi N, Tesler G (2013). QUAST: quality assessment tool for genome assemblies. Bioinformatics.

[30] Low AJ, Koziol AG, Manninger PA, Blais B, Carrillo CD (2019). ConFindr: rapid detection of intraspecies and cross-species contamination in bacterial whole-genome sequence data. PeerJ.

[31] Achtman M, Wain J, Weill F-X, Nair S, Zhou Z (2012). Multilocus sequence typing as a replacement for serotyping in *Salmonella enterica*. PLoS Pathog.

[32] Jolley KA, Bliss CM, Bennett JS, Bratcher HB, Brehony C (2012). Ribosomal multilocus sequence typing: universal characterization of bacteria from domain to strain. Microbiology.

[33] Petkau A, Mabon P, Sieffert C, Knox NC, Cabral J (2017). SNVPhyl: a single nucleotide variant phylogenomics pipeline for microbial genomic epidemiology. Microb Genom.

[34] Page AJ, Cummins CA, Hunt M, Wong VK, Reuter S (2015). Roary: rapid large-scale prokaryote pan genome analysis. Bioinformatics.

[35] Brynildsrud O, Bohlin J, Scheffer L, Eldholm V (2016). Rapid scoring of genes in microbial pan-genome-wide association studies with scoary. Genome Biol.

[36] Fu Y, M’ikanatha NM, Dudley EG (2023). Whole-genome subtyping reveals population structure and host adaptation of *Salmonella* Typhimurium from wild birds. J Clin Microbiol.

[37] Altschul SF, Gish W, Miller W, Myers EW, Lipman DJ (1990). Basic local alignment search tool. J Mol Biol.

[38] Zhou Z, Alikhan N-F, Mohamed K, Fan Y, Achtman M (2020). The EnteroBase user’s guide, with case studies on *Salmonella* transmissions, *Yersinia pestis* phylogeny, and *Escherichia* core genomic diversity. Genome Res.

[39] Olm MR, Brown CT, Brooks B, Banfield JF (2017). dRep: a tool for fast and accurate genomic comparisons that enables improved genome recovery from metagenomes through de-replication. ISME J.

[40] Galili T (2015). Dendextend: an R package for visualizing, adjusting and comparing trees of hierarchical clustering. Bioinformatics.

[41] Paradis E, Schliep K (2018). ape 5.0: an environment for modern phylogenetics and evolutionary analyses in R. Bioinformatics.

[42] Yu G, Smith DK, Zhu H, Guan Y, Lam T-Y (2017). ggtree: an R package for visualization and annotation of phylogenetic trees with their covariates and other associated data. Methods Ecol Evol.

[43] Wickham H, Chang W, Henry L, Pendersen T, Takahashi K (2016). Ggplot2: Elegant Graphics for Data Analysis.

[44] Ido N, Lee K, Iwabuchi K, Izumiya H, Uchida I (2014). Characteristics of *Salmonella enterica* serovar 4,[5],12:i:- as a monophasic variant of serovar Typhimurium. PLoS One.

[45] Brunet YR, Khodr A, Logger L, Aussel L, Mignot T (2015). H-NS silencing of the *Salmonella* pathogenicity island 6-encoded type VI secretion system limits *Salmonella enterica* serovar Typhimurium interbacterial killing. Infect Immun.

[46] Alcoforado Diniz J, Liu YC, Coulthurst SJ (2015). Molecular weaponry: diverse effectors delivered by the type VI secretion system. Cell Microbiol.

[47] Sana TG, Flaugnatti N, Lugo KA, Lam LH, Jacobson A (2016). *Salmonella* Typhimurium utilizes a T6SS-mediated antibacterial weapon to establish in the host gut. Proc Natl Acad Sci U S A.

[48] Silva-Valenzuela CA, Molina-Quiroz RC, Desai P, Valenzuela C, Porwollik S (2016). Analysis of two complementary single-gene deletion mutant libraries of *Salmonella* Typhimurium in intraperitoneal infection of BALB/c mice. Front Microbiol.

[49] Liu J, Guo J-T, Li Y-G, Johnston RN, Liu G-R (2013). The type VI secretion system gene cluster of *Salmonella* Typhimurium: required for full virulence in mice. J Basic Microbiol.

[50] Ogunremi D, Blais B, Huang H, Wang L, Elmufti M (2017). Draft genome sequences of two strains of *Salmonella enterica* serovar Typhimurium displaying different virulence in an experimental chicken model. Genome Announc.

[51] Perez KJ, Martins FS, Cara DCM, Nicoli JR, Tondo EC (2012). Evaluation of intestinal invasion in germ‐free mice challenged with acid‐adapted and nonacid‐adapted *Salmonella enteritidis* SE86 and *Salmonella* Typhimurium ST99. J Food Saf.

[52] Reuven NB, Tomer G, Livneh Z (1998). The mutagenesis proteins UmuD’ and UmuC prevent lethal frameshifts while increasing base substitution mutations. Mol Cell.

[53] Smith C, Eisenstadt E (1989). Identification of a umuDC locus in *Salmonella* Typhimurium LT2. J Bacteriol.

[54] Nohmi T, Yamada M, Watanabe M, Murayama SY, Sofuni T (1992). Roles of *Salmonella* Typhimurium umuDC and samAB in UV mutagenesis and UV sensitivity. J Bacteriol.

[55] Koch WH, Kopsidas G, Meffle B, Levine AS, Woodgate R (1996). Analysis of chimeric UmuC proteins: identification of regions in *Salmonella* Typhimurium UmuC important for mutagenic activity. Mol Gen Genet.

[56] Zhang S, Den Bakker HC, Li S, Chen J, Dinsmore BA (2019). SeqSero2: rapid and improved *Salmonella* serotype determination using whole-genome sequencing data. Appl Environ Microbiol.

